# Use of antidepressant medications among older adults in European long-term care facilities: a cross-sectional analysis from the SHELTER study

**DOI:** 10.1186/s12877-020-01730-5

**Published:** 2020-08-27

**Authors:** Silvia Giovannini, Graziano Onder, Henriëtte G. van der Roest, Eva Topinkova, Jacob Gindin, Maria Camilla Cipriani, Michael D. Denkinger, Roberto Bernabei, Rosa Liperoti, on behalf of the SHELTER Study Investigators

**Affiliations:** 1 Department of Aging, Neurological, Orthopaedic and Head-Neck Sciences, Fondazione Policlinico Universitario Agostino Gemelli IRCCS, Rome, Italy; 2 Istituto Superiore di Sanità, Rome, Italy; 3 Department of General Practice and Elderly Care Medicine, Amsterdam Public Health Research Institute, VU University Medical Center, Amsterdam, The Netherlands; 4 Department of Geriatrics, First Faculty of Medicine, Charles University, Prague, Czech Republic; 5 The Centre for Standards in Health and Disability, Research Authority, University of Haifa, Haifa, Israel; 6 AGAPLESION Bethesda Clinic, Competence Centre of Geriatrics, University of Ulm, Ulm, Germany; 7 Fondazione Policlinico Universitario A. Gemelli IRCCS – Università Cattolica del Sacro Cuore, Rome, Italy

**Keywords:** Older adults, Antidepressants, Nursing homes

## Abstract

**Background:**

Late-life depression is common among older adults living in nursing homes (NHs). Over the last 30 years there has been an increase in the rates of prescription of antidepressant medications across all ages, with the largest rise reported in older adults. This study aimed to describe the pattern of antidepressant medication use among NH residents from 7 European countries and Israel and to examine patient and facilities characteristics that may account for it.

**Methods:**

We conducted a cross-sectional analysis of data from the SHELTER study, an observational longitudinal cohort study that collected comprehensive resident data using the interRAI Long-Term Care Facility instrument in 7 European Countries and Israel.

Descriptive statistics were used to examine sample characteristics. Potential correlates of antidepressant medication use were identified using multiple logistic regression modeling.

**Results:**

Among 4023 residents entering the study, 32% had depressive symptoms and nearly half of these individuals used antidepressants. Antidepressant medication use varied by country, with a prevalence in the overall sample of 35.6% (*n* = 1431). Among antidepressant users, 59.9% were receiving selective serotonin reuptake inhibitors (SSRI). The strongest correlates of antidepressant use included reported diagnosis of anxiety, depression, bipolar disorder, pain, falls and high level of social engagement. Age over 85 years, living in facilities located in rural areas and a diagnosis of schizophrenia reduced the likelihood of being prescribed with an antidepressant.

**Conclusions:**

A large proportion of residents in European long-term care facilities receive antidepressant medications. The decision to prescribe antidepressants to NH residents seems to be influenced by both patient and facility characteristics. Future longitudinal studies should evaluate the efficacy and safety of antidepressant use in NHs thus providing evidence for recommendations for clinical practice.

## Background 

Late-life depression is one of the most common psychiatric disorder in the elderly population, affecting up to 30% of those 65 years and older [1, 2] and it has been associated with increased morbidity [3] and mortality [4]. The prevalence of depression varies in different settings from 10% among community-dwelling elderly [5] individuals to as much as 35% among nursing home (NH) residents [6]. Such high prevalence among institutionalized individuals has been related to the high rates of physical comorbidity coupled with factors such as family disconnection, reduced social engagement or environmental changes that are likely to characterize this population [7].

Prescription of antidepressant medications has significantly increased from 21.9% in 1996 to 47.5% in 2006 in US NHs [8] despite concerns related to their safety and appropriateness of psychotropic medications in this population. In fact, the course of depression may be heterogeneous, and treatment should be tailored to patients’ individual needs. Several age-related factors and comorbidities complicate pharmacotherapy among NH residents who use more medications than patients in any other setting because of the number and severity of chronic diseases. However, depression is an independent risk factor for all-cause mortality in nursing homes [9] and it has been suggested that underuse of antidepressants is associated with increased disability, worsening of clinical outcomes and increased mortality [10]. Present guidelines recommend antidepressants and specifically selective serotonin reuptake inhibitors (SSRIs) as the first-line treatment of depression in older patients [11, 12]. Previous studies have documented that prescription rates of psychotropic medications in NHs may be influenced by facility factors including the presence of a professional geriatrician [13].

The aim of this pharmacoepidemiological study was to describe the pattern of use of antidepressant medications in a sample of NH residents in 7 European countries and Israel and to identify individual socio-demographic and clinical characteristics as well as facility-related factors that correlate with their use.

## Methods

### Data source

Data for this cross-sectional study was obtained from the Services and Health for Elderly in Long TERm care (SHELTER) study, a project funded by Seventh Framework Programme of the European Union. Methodology of the SHELTER study is described in detail elsewhere [14]. The study was conducted during the years 2009 to 2011. Briefly, older adults residing in participating nursing homes at the beginning of the study and those admitted in the 3-month enrolment period following the initiation of the study were assessed using the interRAI instrument for Long-Term Care (interRAI LTCF) at baseline and reassessed at 6 and 12 months (follow-up assessments) if still residing in the facility. If no longer in the facility, reason (death, hospitalization, discharged to home or to another institution) and date of death or discharge were recorded. The only exclusion criterion was unwillingness to participate to the study. Study partners of the SHELTER project identified NHs willing to participate in the study. Patients residing in participating NHs at the initiation of the study and those who were admitted during the following 3-month period were assessed by use of the interRAI-LTCF tool. Overall, 4156 residents were included from 57 NHs across 7 European countries (10 NHs in the Czech Republic, 9 in England, 4 in Finland, 4 in France, 9 in Germany, 10 in Italy, 4 in The Netherlands) and 1 Non-European country (7 in Israel). The aim of the SHELTER study was to assess the use of the interRAI-LTCF, when translated into the languages of participating countries, as a tool to collect information about residents and to assess their care needs and the provision of care in NHs in EU countries. Overall, 197 of the 198 the interRAI-LTCF items tested met or exceeded standard cut-offs for acceptable test-retest and inter-rater reliability [14].

### Ethics and consent to participate

Ethical approval to conduct the study was given by the Ethics and Research Committee of Catholic University of the Sacred Heart of Rome. Ethical approval was obtained also from the ethics committees of the participating centres. All procedures performed in this study involving human participants were in accordance with the ethical approval and standards of the local ethics committees. Written informed consent was obtained from all participants.

### Study sample

The SHELTER database was the source data for the current study. It includes data on 4156 residents assessed with interRAI LTCF. From such initial sample, we excluded only those residents with missing drug data (133 residents; 3.2% of the overall sample) leading to a final sample of 4023 residents entering the study.

### Antidepressant use

Drugs were classified by the Anatomical Therapeutic Chemical (ATC) classification system controlled by the World Health Organization Collaborating Centre for Drugs Statistics Methodology (WHOCC). This system classifies the active ingredient of a drug into groups according to the organ or system on which they have their effect.

In the SHELTER study medication use was defined as use in the 3-day period prior to the interRAI LTCF assessment. Based on the ATC classification, antidepressants were classified as follows: TCA (ATC: N06AA), SSRI (ATC: N06AB), SNRI (ATC: N06AX16 and N06AX21), serotonin modulators (ATC: N06AX05) and other antidepressants (ATC: others including N06A). Patients receiving antidepressants from two or more different classes were placed in a separate group.

### Covariates

Independent variables included in the model are demographic factors age and gender. Facility characteristics included are area (urban or rural), number of residential beds, presence of a Dementia Care Unit and whether amongst staffing geriatricians, pharmacists, or psychiatrists were present. A variable indicating the specific Country was also included.

Residents´ characteristics were collected by the interRAI LTCF. The InterRAI LCTF is a standardized assessment instrument designed to assess care needs and preferences of NH residents and to assist with care planning. It contains more than 350 items that comprehensively capture client characteristics, including sociodemographic variables, clinical characteristics including both physical and cognitive status, clinical diagnoses, current services, and medication use.

Behavioral symptoms included wandering, verbal abuse, physical abuse, socially inappropriate or disruptive behavior, inappropriate public sexual behavior or public disrobing and resistance of care. Psychotic symptoms were present if the patients scored positive for the presence of hallucinations, delusions or both.

The Activities of Daily Living (ADL) Hierarchy scale was used to determine functional impairment with levels varying from 0 (no impairment) to 6 (total dependence). Cognitive impairment was determined by use of the Cognitive Performance scale (CPS) with scores ranging from 0 (intact) to 6 (very severe impairment) [15]. In the model both scale outcome scores were categorized normal to mild (CPS or ADL Hierarchy scale score 0–1), moderate (CPS or ADL Hierarchy scale score 2–4) and severe impairment (CPS or ADL Hierarchy scale score ≥ 5). The level of social engagement was estimated using the Revised Index for Social Engagement (RISE), ranging from 0 to 6, a higher score indicating higher social engagement. Scale outcome scores were categorized low (RISE score 0–1), medium (RISE score 2–4) and high (RISE score ≥ 5). For the presence of depressive symptoms, patients were screened using the Depression Rating scale with cutoff-point score ≥ 3 out of 7. Compared to DSM-IV major or minor depression diagnoses, the DRS has been shown 91% sensitive and 69% specific at a cut-point score of 3 out of 7 [16]. Pain was defined as either complaining about or showing evidence of pain in the last 3 days or not shown in the last 3 days before assessment, but present before then. Falls were defined as the occurrence of a fall in the 90 days prior to the assessment. Diagnoses reported in the InterRAI LTCF were based on consultation of medical records and collection of medical history.

### Statistical analysis

Sample characteristics have been reported as mean, standard deviation (SD) or rate (%), as appropriate. Two-tailed T test was used to compare continuous variables according to use of antidepressants. Chi-squared test was used to compare categorical variables. Multiple logistic regression modeling was used to examine the association between the independent variables and use of antidepressants.

Independent variables included in the analysis were age, gender and variables associated with the use of antidepressants at the univariate analysis at a *p* value < 0.10. An additional logistic regression was performed among residents receiving antidepressants (*n* = 1431) to assess variables independently associated with use of SSRI. Both models were adjusted for country. All analyses were conducted using SAS software (version 8.2; SAS Institute, Inc., Cary, NC).

## Results

Mean age of 4023 residents in the study was 83.6 years (± 9.4 years) and 73.2% were women. Of all residents, nearly 32% had depressive symptoms (DRS ≥ 3) and 47.2% (*n* = 598) of them used antidepressant medications.

**Fig. 1:**
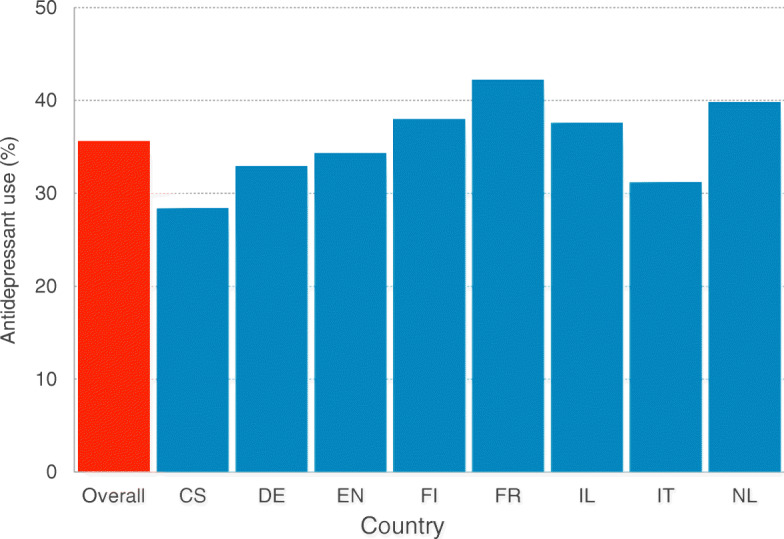
Prevalence of antidepressant drugs use among nursing home residents, by country. The SHELTER Study (*n* = 1431) CS = Czech Republic; DE = Germany; EN: England; FI=Finland; FR = France; IL = Israel; IT = Italy; NL = Netherlands; SHELTER = Services and Health for Elderly in Long Term care

**Fig. 2:**
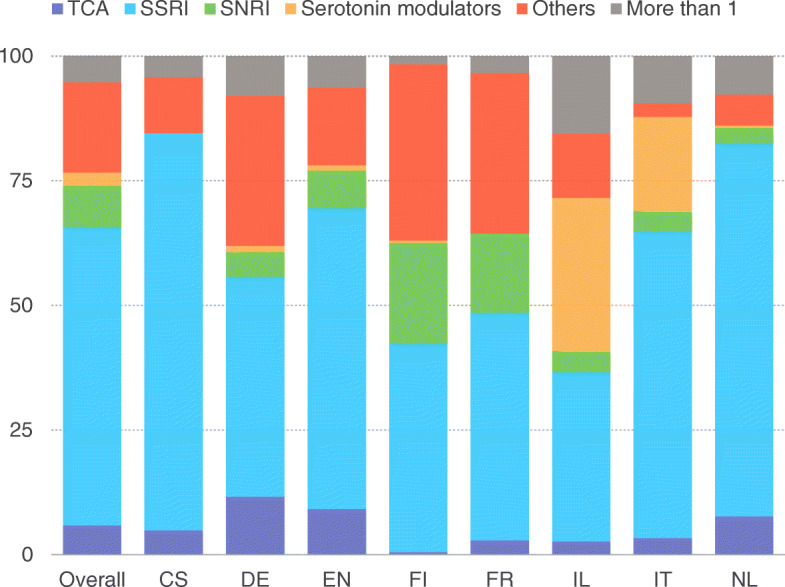
Percentage of nursing home residents receiving most commonly prescribed antidepressants

Antidepressant medication use in the overall sample was 35.6% (*n* = 1431) ranging from 28.4% (Czech Republic) to 42.2% (France) (Fig. 1). Among antidepressant users, 59.9% were treated with selective serotonin reuptake inhibitors (SSRIs), 8.2% were treated with serotonin–norepinephrine reuptake inhibitors (SNRIs), 5.9% were treated with tricyclic antidepressants (TCAs), 2.6% treated with serotonin modulators, 18.2% were receiving antidepressants from other classes and 5.4% were on multiple antidepressants (Fig. 2). Of residents receving SSRIs, citalopram was most commonly used (61.0%), followed by sertraline (13.6%), escitalopram (11.9%), and paroxetine (10.1%). Those using SNRIs received venlafaxine (76.7%) or duloxetine (23.3%). Among residents using TCAs, amitriptyline was most commonly used (57.3%), followed by mianserin (16.3%) and tianeptine (4.0%). The only serotonin modulator used was trazodone wich had a high prevalence of use in Israel (30.7%) and in Italy (18.9%).

Table 1 shows the baseline characteristics of residents, according to antidepressant medication use. Residents using antidepressants were slightly younger, more often in urban areas, in facilities with a demenia care unit, where a pharmacist or psychiatrist were present, as compared with those not using antidepressants. Moreover, the group using antidepressants had more psychotic symptoms, was less functionally impaired and showed higher levels of social engagement. Among reported clinical diagnoses, depression, anxiety, bipolar disorder and sleep disturbances were more common among those residents on antidepressants compared to those not using these medications.

**Table 1:** Baseline Demographic and Clinical Characteristics of Residents and Facility factors by Antidepressant Drug Use^a^

	Antidepressant Use		
	No (*n* = 2592)	Yes (*n* = 1431)	*p*-value
*Demographic factors*			
Age			
< 65	112 (4,3)	64 (4,5)	**0,02**
≥ 65–75	286 (11,0)	186 (13,0)	
≥ 75–85	869 (33,5)	509 (35,6)	
≥ 85	1325 (51,1)	672 (47,0)	
Female gender	1877 (72,4)	1068 (74,6)	0,13
*Facility factors*			
Area			
Urban	2053 (79,2)	1183 (82,7)	**0,008**
Rural	539 (20,8)	248 (17,3)	
Residential beds			
0–49	244 (9,4)	183 (12,8)	0,63
50–99	368 (14,2)	187 (13,1)	
100–199	1294 (49,9)	581 (40,6)	
≥ 200	686 (26,5)	480 (33,5)	
Dementia Care Unit	1877 (86,8)	1088 (89,8)	**0,01**
Geriatrician	1194 (46,1)	681 (47,6)	0,35
Pharmacist	543 (20,9)	364 (25,4)	**0,001**
Psychiatrist	664 (25,6)	428 (29,9)	**0,003**
*Patient characteristics*			
Behavioral symptoms	536 (20,7)	333 (23,3)	0,06
Psychotic symptoms	233 (9,0)	178 (12,4)	**0,001**
Functional impairment			
Mild	456 (17,6)	288 (20,2)	**0,02**
Moderate	1066 (41,2)	595 (41,7)	
Severe	1063 (41,1)	544 (38,1)	
Cognitive impairment			
None/Mild	760 (29,8)	461 (32,5)	0,06
Moderate	941 (36,9)	569 (40,1)	
Severe	846 (33,2)	388 (27,4)	
Social engagement			
Low	803 (31,5)	341 (24,0)	**< 0,001**
Med	699 (27,4)	453 (31,9)	
High	1048 (41,1)	625 (44,0)	
*Symptoms and Diagnoses*			
Depressive symptoms	670 (26,3)	598 (42,2)	**< 0,001**
Anxiety	218 (8,4)	243 (17,1)	**< 0,001**
Schizophrenia	96 (3,7)	28 (2,0)	**0,002**
Bipolar disorder	37 (1,4)	33 (2,3)	**0,04**
Sleep disorder	553 (21,4)	377 (26,5)	**< 0,001**
Pain	834 (32,3)	614 (43,0)	**< 0,001**
Falls	436 (17,0)	310 (22,0)	**< 0,001**
Stroke/CVA	559 (21,7)	327 (23,0)	0,34
Coronary heart disease	685 (26,6)	365 (25,7)	0,55
Congestive heart failure	445 (17,2)	263 (18,5)	0,31
COPD	236 (9,1)	134 (9,4)	0,77
Cancer	262 (10,1)	173 (12,1)	0,05

^a^ overall number of residents 4023; overall number of nursing homes 57

Table 2. shows results from the multivariate logistic regression model examining potential correlates of antidepressant use. The likelihood of receiving antidepressants was lower for those 85 years of age or older (odds ratio (OR) 0.68; 95% Confidence Interval (CI) 0.47–0.99). There was no significant difference with respect to gender. Facilities located in a rural area were associated with lower antidepressant use than those in urban areas (OR = 0.66; 95%CI 054–0.81). Both medium and high level of social engagement were associated with higher use of antidepressants (OR = 1.52; 95%CI 1.23–1.87 and OR = 1.40; 95%CI 1.13–1.74, respectively). Presence of pain increased the likelihood of being prescribed with antidepressants (OR = 1.35; 95%CI 1.16–1.59). An increased likelihood of falling was also associated with antidepressant use (OR 1.25; 95% CI 1.03–1.51) Finally, a strong association with antidepressant use was found with depression (OR = 1.78; 1.51–2.10), and anxiety (OR 1.93; 95%CI 1.50–2.49).

**Table 2:** Factors associated with antidepressant use: results from the multivariate logistic regression model (reference category: no use)

Characteristics	Odds Ratio a	95% C.I.
*Demographic factors*		
Age < 65	–	–
≥ 65–75	1,02	0,68-1,54
≥ 75–85	0,83	0,57-1,21
**≥ 85**	**0,68**	**0,47-0,99**
Female gender	1,14	0,96-1,37
*Facility factors*		
**Rural area**	**0,66**	**0,54-0,81**
**Dementia Care Unit**	**1,31**	**1,03-1,67**
**Pharmacist**	**1,43**	**1,19-1,72**
Psychiatrist	1,06	0,89-1,27
*Patient characteristics*		
Behavioral symptoms	1,00	0,83-1,21
Psychotic symptoms	1,21	0,95-1,55
Functional impairment		
Moderate	0,95	0,76-1,18
Severe	1,09	0,85-1,40
Cognitive impairment		
Medium	0,90	0,74-1,10
High	0,78	0,61-1,00
Social engagement		
**Medium**	**1,52**	**1,23-1,87**
**High**	**1,40**	**1,13-1,74**
*Symptoms and Diagnoses*		
**Depressive symptoms**	**1,78**	**1,51-2,10**
**Anxiety**	**1,93**	**1,50-2,49**
**Schizophrenia**	**0,34**	**0,20-0,56**
Bipolar disorder	1,77	1,00-3,14
Sleep disorder	1,10	0,93-1,31
**Pain**	**1,35**	**1,16-1,59**
**Falls**	**1,25**	**1,03-1,51**
Cancer	1,13	0,89-1,44

^a^ All data are adjusted for country. Overall number of residents 4023; overall number of nursing homes 57

A second multivariate logistic model explored factors associated with the use of SSRI versus other antidepressants (see Table 3). Facility characteristics including both the presence of a pharmacist and the presence of psychiatrist appeared to influence the choice of prescribing an SSRI agent compared to other antidepressants (OR 0.64; 95%CI 0.47–0.87, and OR 1.86; 95% CI 1.38–2.50, respectively). Finally, the presence of a sleep disorder decreased the likelihood of being treated with SSRI in favor of other classes of antidepressants (OR 0.64; 95%CI 0.48–0.85).

**Table 3:** Factors associated with SSRI use: results from the multivariate logistic regression model (reference category: other antidepressants)

Characteristics	Odds Ratio a	95% C.I.
*Demographic factors*		
Age < 65	–	–
≥ 65–75	0,70	0,34-1,46
≥ 75–85	0,62	0,31-1,24
≥ 85	0,51	0,26-1,01
Female gender	1,02	0,75-1,39
*Facility factors*		
Rural area	1,11	0,77-1,60
Dementia Care Unit	0,69	0,45-1,08
Pharmacist	0,64	0,47-0,87
**Psychiatrist**	**1,86**	**1,38-2,50**
*Patient characteristics*		
Behavioral symptoms	0,87	0,64-1,19
Psychotic symptoms	0,91	0,62-1,35
Functional impairment		
Moderate	0,81	0,55-1,18
Severe	1,35	0,88-2,07
Cognitive impairment		
Medium	1,07	0,76-1,51
High	0,78	0,52-1,18
Social engagement		
Medium	0,98	0,69-1,39
High	1,19	0,83-1,71
*Symptoms and diagnoses*		
Depressive symptoms	0,85	0,65-1,12
Anxiety	0,80	0,55-1,16
Schizophrenia	0,42	0,16-1,08
Bipolar disorder	0,98	0,42-2,26
Sleep disorder	0,64	0,48-0,85
Pain	0,89	0,69-1,16
Falls	1,36	0,98-1,87
Cancer	1,00	0,67-1,48

^a^ All data are adjusted for country. Overall number of residents 4023; overall number of nursing homes 57

## Discussion

In the present study we have documented that almost one third of NH residents have depressive symptoms. However, although use of antidepressants is quite common in the general NH population (35.6%), less than half of residents with documented depressive symptoms are treated with antidepressant medications. Such findings are in line with previously reported prevalence rates of antidepressant use in NHs ranging from 35 to 60% [17] with only half of the residents with diagnosed depression receiving antidepressant treatment [18]. The discrepancy documented in long-term care setting between antidepressant prescriptions and rate of depression diagnoses has been variously interpreted [12]. In fact, this finding may be related to the different indications for using antidepressant medications, including for example anxiety, agitation, sleep disorders, and pain. In our study, a quite strong correlation has been found between antidepressant use and other conditions namely anxiety, bipolar disorder and pain. A possible under-prescription of antidepressants to residents with depressive symptoms may be also explained by suboptimal treatment effectiveness of these medications among frail multimorbid individuals with frequent cognitive and functional impairment [19]. Also, physicians may be reluctant to prescribe antidepressant medications to NH residents who usually present with multiple diseases and polypharmacy and are likely to develop adverse drug reactions [20]. Indeed, among residents in our sample, those who were younger and less functionally and cognitively impaired were more likely to receive an antidepressant as compared to the other individuals.

Among different classes of antidepressant medications, SSRI accounted for most prescriptions in our study in all included countries. Such finding was consistent with previous data. For example, in a large sample of Veterans Health Administration NH residents, over half of them received antidepressants and SSRI accounted for 54.3% of the prescriptions [21]. Similar findings were reported from a Norwegian study where 40% of NH residents were treated with antidepressants, 50% of which were SSRIs [22]. Moreover, SSRI were ranked in the top 20 drug classes that accounted for nearly 70% of total drug costs for long-stay NH residents of 136 facility throughout United States [21]. Our work reported that SSRI use in the overall study sample was nearly tenfold higher than that of tricyclic antidepressants. The evidence that SSRIs are the treatment of choice for depression in the elderly population is growing [23]. Moreover, numerous safety issues regarding the use of TCAs in elderly individuals limit the use of this class in such population. However, it is worth to mention that also the safety profile of SSRIs in the elderly population has been recently questioned and some potential important side effects including falls, hyponatremia and stroke may actually limit their use in this population [20].

We documented that antidepressant prescribing pattern was quite heterogeneous across the eight countries. For instance, in Israel serotonin modulators were prescribed almost in the same proportion as SSRI and prescription of multiple antidepressants in combination was the highest of the all sample. In Czech Republic the use of neither serotonin–norepinephrine reuptake inhibitors nor serotonin modulators was reported. Part of such variation may be explained by differences in country-specific cost-containment policies with respect to antidepressants, including preferred drug lists. Moreover, consistent with the US scenario [24]. drug policies, copayment systems, drug utilization recommendations, and physician and patient education may largely vary by country and impact substantially on the prescription choice.

According to our findings, the very old residents are the least likely to be prescribed antidepressant medications. This is consistent with previous studies showing a decrease in antidepressant use with age, especially among those 85 years or older [25]. In our study, residents from facilities in rural areas were less likely to receive antidepressants compared with those in urban areas. This finding replicates evidence from a previous study that reported higher prevalence of antidepressant use among urban compared to rural NH residents [26]. We also found that the use of antidepressants was significantly associated with high levels of social engagement. It is possible that residents who were more socially engaged had a higher compliance with treatments and better access to care. Also, appropriately controlled depressive symptoms increases social involvement as reported from Kang who documented that depression was the strongest factor affecting social engagement among NH residents with dementia [27]. Finally, such findings may indicate that the need for antidepressant treatment may be unrecognized among those residents who are most likely to be overlooked such as the oldest and the least socially engaged ones.

Although antidepressants are mostly prescribed for the treatment of depression and other psychiatric conditions, there might a be a substantial amount of residents that were treated for reasons other than conventional indications. In our sample, pain was strongly associated with antidepressant use. This finding is not surprising, given that several antidepressants such as duloxetine and amitriptyline are effective on chronic or neuropathic pain, and anxiety and depression are often associated with experiencing pain through inhibition of modulatory pathways [28]. A recent paper has documented a significant increase in prescription of analgesic and antidepressant medications after NH admission [29], being the activity restriction experienced by residents a possible mechanism of increased risk of depression [30]. Residents with a history of falls in the last 90 days were more likely to use antidepressants compared with non fallers. Antidepressant medications may cause or worsen orthostatic hypotension and have specifically been found to predispose elderly patients to falling [31].

Of the examined variables related to the facilities, the presence of a dementia care unit and/or the provision of a pharmacist or a psychiatrist, increased the likelihood to receive an antidepressant. The availability of a psychiatric care service also increased the likelihood of receiving SSRIs compared with other classes of antidepressant medications. One possible explanation of the observed findings may be related to the fact that the presence of such services may reflect specialists´ input and thoughtful drug selection. It could be also argued that the presence of a psychiatrist may be related to a specific case-mix of the facility for example with prevalent psychopathological morbidity.

Among other clinical factors investigated, only the presence of a sleep disorder appeared to influence the choice of a specific class of antidepressant medications reducing the likelhood of receiving SSRIs. This finding may be related to the described sedative properties of several compounds, such as trazodone and mirtazapine that may be perceived as the best choice for treating depressive symptoms associated with sleep disturbances.

### Strengths and limitations

This was a large, multinational study that could provide a picture of antidepressant use in Europe and Israel. However, the study has some limitations. No inference on cause-effect relationship can be made because of the cross-sectional design of the study. Although we have examined numerous individual and facility characteristics, several residual factors related to resident case-mix and structural and organizational aspects may have influenced the frequency and pattern of antidepressant prescription. In the present study we could reliably identify depressive symproms using the DRS scale but we could not measure the prevalence of depression. In fact, although DRS showed a good correlation with clinical criteria, it should not be intended as an instrument to diagnose depression. Morever, the DRS is not intended to measure the severity of depressive symptoms and therefore we could not explore a possible differential probability of being prescribed with antidepressants according to the degree of disease severity. Several non-pharmacological options are known to be effective tratments for depression. However we did not explore the frequency of such treaments. No information was available to measure the frequency of alternative indications for antidepressant treatment such as other psychiatric disorders, neuropsychiatric symptoms of dementia or sleep disorders. Facilities included in the study were not randomly selected thus they may not be nationally representative nor the results generalizable to all NH residents in each country.

## Conclusion

The present study showed that depressive symptoms are frequent among NH residents in Europe and Israel although less than half of resident with depressive symptoms receive pharmachological antidepressant treatment. The need for antidepressant treatment may be especially unrecognized among the oldest and least socially engaged residents. The use of antidepressant medications is also quite common and they are possibly used for conditions other than conventional indications in many cases. Future longitudinal studies will have to assess the risk-to -benefit ratio associated with the use of antidepressant medications in such fragile population thus providing evidence to inform clinical recommendations and to guide prescribing choices.

## Supplementary information

**Additional file 1.**

## Data Availability

The data that support the findings of this study are available from the Shelter project investigators but restrictions apply to the availability of these data, which were used under license for the current study, and so are not publicly available. Data are however available from the authors upon reasonable request and with permission of all Shelter project investigators.

## Abbreviations

NHs: Nursing Homes
SSRI: selective serotonin reuptake inhibitors
SHELTER: Services and Health for Elderly in Long TERm care
interRAI-LTCF: interRAI instrument for Long-Term Care
ADL: Activities of Daily Living
CPS: Cognitive Performance scale
RISE: Revised Index for Social Engagement
SD: Standard Deviation
TCAs: Tricyclic Antidepressants
OR: Odds Ratio
CI: Confidence Interval
