# Supplementary material for: Use of antidepressant medications among older adults in European long-term care facilities: a cross-sectional analysis from the SHELTER study
Source: BMC Geriatr. 2020 Aug 27;20:310. doi: 10.1186/s12877-020-01730-5 (PMC7457305; doi:10.1186/s12877-020-01730-5)
Supplement: Supplementary file 1 — Additional file 1. [file 12877_2020_1730_MOESM1_ESM.docx]

**Appendix.**

**Classification Antidepressants ATC codes**

TCA (N06AA)
 N06AA04 : Clomipramine
 N06AA05 : Opipramol
 N06AA06 : Trimipramine
 N06AA07 : Lofepramine
 N06AA09 : Amitriptyline
 N06AA10 : Nortriptyline
 N06AA12 : Doxepin
 N06AA16 : Dosulepin
 N06AA21 : Maprotiline

SSRI (N06AB)
 N06AB03 : Fluoxetine
 N06AB04 : Citalopram
 N06AB05 : Paroxetine
 N06AB06 : Sertraline
 N06AB08 : Fluvoxamine
 N06AB10 : Escitalopram

SNRI
 N06AX16 : Venlafaxine
 N06AX21 : Duloxetine

Serotonin modulators
 N06AX05 : Trazodone

Others
 N06AG02 : Moclobemide

 N06AX03 : Mianserin
 N06AX11 : Mirtazapine
 N06AX12 : Bupropion
 N06AX14 : Tianeptine
 N06AX17 : Milnacipran
 N06AX18 : Reboxetine
